# Could Cord Blood Cell Therapy Reduce Preterm Brain Injury?

**DOI:** 10.3389/fneur.2014.00200

**Published:** 2014-10-09

**Authors:** Jingang Li, Courtney A. McDonald, Michael C. Fahey, Graham Jenkin, Suzanne L. Miller

**Affiliations:** ^1^The Ritchie Centre, MIMR-PHI Institute, Clayton, VIC, Australia; ^2^Department of Paediatrics, Monash University, Clayton, VIC, Australia; ^3^Department of Obstetrics and Gynaecology, Monash University, Clayton, VIC, Australia

**Keywords:** preterm birth, low birth weight, brain damage, white matter injury, oligodendrocytes, cerebral palsy, umbilical cord blood, stem cells, hypoxia–ischemia, inflammation, periventricular leukomalacia

## Abstract

Major advances in neonatal care have led to significant improvements in survival rates for preterm infants, but this occurs at a cost, with a strong causal link between preterm birth and neurological deficits, including cerebral palsy (CP). Indeed, in high-income countries, up to 50% of children with CP were born preterm. The pathways that link preterm birth and brain injury are complex and multifactorial, but it is clear that preterm birth is strongly associated with damage to the white matter of the developing brain. Nearly 90% of preterm infants who later develop spastic CP have evidence of periventricular white matter injury. There are currently no treatments targeted at protecting the immature preterm brain. Umbilical cord blood (UCB) contains a diverse mix of stem and progenitor cells, and is a particularly promising source of cells for clinical applications, due to ethical and practical advantages over other potential therapeutic cell types. Recent studies have documented the potential benefits of UCB cells in reducing brain injury, particularly in rodent models of term neonatal hypoxia–ischemia. These studies indicate that UCB cells act via anti-inflammatory and immuno-modulatory effects, and release neurotrophic growth factors to support the damaged and surrounding brain tissue. The etiology of brain injury in preterm-born infants is less well understood than in term infants, but likely results from episodes of hypoperfusion, hypoxia–ischemia, and/or inflammation over a developmental period of white matter vulnerability. This review will explore current knowledge about the neuroprotective actions of UCB cells and their potential to ameliorate preterm brain injury through neonatal cell administration. We will also discuss the characteristics of UCB-derived from preterm and term infants for use in clinical applications.

## Background

Impressive advances in perinatal and neonatal care have led to substantial improvements in survival rates for preterm infants born at <37 weeks gestation. However, survival of preterm infants may occur at a cost, with a strong causal link between preterm birth and subsequent neurological motor and cognitive deficits, including cerebral palsy (CP). In particular, more extremely preterm babies now survive than ever before with these infants at the greatest risks of short and long-term neurodevelopmental deficits (1). Despite the known etiological link between preterm birth and neuro-motor and neuro-cognitive dysfunctions, there are currently no specific neuroprotective treatments available for preterm infants.

Stem, or stem-like cells, have drawn attention from scientists and the general public due to their potential to induce tissue repair and/or regeneration. Umbilical cord blood (UCB)-derived cells offer ethical and practical advantages over other stem-like cells given that collection can be obtained from the discarded placenta at birth. Such cells possess multiple proven [as per cord blood hematopoietic stem cells (HSCs)] and potential therapeutic uses, including recent evidence that UCB cells may mitigate newborn brain damage arising from term neonatal hypoxic-ischemic encephalopathy (HIE). However, despite the heightened neurological risks associated with preterm births, the potential use of UCB cells in preterm neonates has not yet been actively investigated. This article briefly describes the background, etiology, and pathophysiological mechanisms of brain injury in preterm infants, and summarizes current research on the use of UCB cells for therapeutic use in term and preterm perinatal brain injury. Potential implications for future clinical trials of UCB cell therapy in preterm infants are discussed.

## Preterm Birth and Childhood Neurological Deficits

In 2010, 14.9 million babies worldwide were born preterm, accounting for approximately 11% of all births, with the rates and burden of preterm birth significantly increased in both low and high-income birth settings compared to the previous decade (2). Of all preterm births in the developed world, 16% are born before 32 weeks of gestation or weigh <1500 g (2), with this population of *very preterm* infants (born 28 to <32 weeks) or *extremely preterm* infants (<28 weeks) at the greatest risks for long-term physical and neurological morbidities. Indeed, in developed countries, preterm births account for 70% of neonatal deaths and up to 75% of neonatal morbidity (3), with the risks of death or disability profoundly increased in middle- or low-income birth settings, reflecting decreased resources for neonatal intensive care (4). In addition, in developed countries most preterm babies now survive as a result of advances in neonatal intensive care such that the survival rate for extremely preterm infants is 90% (2).

Cerebral palsy is the most common physical disability of childhood, occurring in 2–2.5/1000 live births in developed countries. This rate is increased to approximately 90–100/1000 babies that were born at <32 weeks gestation (5, 6). Indeed, 35–50% of children with established CP were born preterm (7, 8). The major overt neurological manifestations of brain injury observed in children that were born preterm are spastic motor deficits, commonly accompanied by intellectual deficits. Less severe disturbances of motility, cognition, and behavior occur in 25–50% of survivors (9).

The economic cost of preterm birth and CP are high due to the need for neonatal intensive care and ongoing long-term complex health care. The National Institute of Medicine estimated that the lifetime cost of all preterm births is $26.2 billion USD per year in the USA (10). The financial burden of CP in the USA has been separately costed and estimated at $11.5 billion USD (11) and is indicative of the large financial burden association with preterm birth and CP. This is in addition to the significant burden placed on families and society who care for children and adults with CP. There is therefore an enormous demand to prevent or reduce brain injury in preterm infants, to reduce the subsequent neurodevelopmental sequelae, and consequently decreasing the large socio-economical burden.

The complications associated with preterm birth and brain injury are complex and involve multiple overlapping adverse pathways, but it is clear that preterm birth is strongly associated with damage to the white matter of the immature brain. Therefore, an understanding of white matter injury (WMI) is a critical component required for the treatment of preterm brain injury.

## White Matter Injury

Fetal brain maturation and functional development involves a series of organizational processes including neurogenesis, cell migration, cell differentiation, synaptogenesis, and axonal myelination. The development of white matter requires mature oligodendrocyte glial cells to produce myelin and ensheath the axons of neurons, and thus oligodendrocytes play a crucial role in fast signal transmission along neurons and throughout the brain. Injury to these cells impairs, usually irreversibly, myelination. Oligodendrocytes develop according to a well-defined lineage. Pre-oligodendrocytes are the predominating oligodendroglial cell at gestational age 24–32 weeks in humans. They are exquisitely vulnerable to pro-inflammatory cytokines, excitotoxicity, oxygen free radical attack, and hypoxic stress, and rapidly undergo apoptosis under adverse conditions (12–15). It is believed that this selective vulnerability of the pre-oligodendrocytes in preterm infants restricts the number and functional ability of mature oligodendrocytes to undergo the process of laying down of white matter and formation of myelin fibers, thus causing very preterm and extremely preterm infants to be most susceptible to WMI (9, 16, 17). Thus, preserving oligodendrocytes and their precursor cells is fundamental to reducing injury to the developing white matter of the brain. Most commonly, preterm brain injury is evident in the periventricular white matter adjacent to the lateral ventricles, so-called periventricular leukomalacia (PVL). WMI is detectable in at least 50% of infants born very preterm or extremely preterm, and is a strong indicator of long-term neurological adverse outcome. Nearly 90% of preterm infants who later develop spastic CP have evidence of WMI (9). Half of the children identified as having WMI will have cognitive and/or behavioral and/or attention deficits (18–20). Clinical imaging studies demonstrate that myelin loss (hypomyelination) and disorganization of major white matter fiber tracts correlate with functional impairments in children with CP and PVL (21, 22).

Pathologically, WMI is a condition demonstrated by coagulation and necrosis of white matter near the lateral ventricles, accompanied by gliosis (23). The periventricular area is vulnerable to ischemia in the preterm brain, which, in part, is anatomically due to poor vascularization and immature cerebrovascular autoregulation (24, 25). This may result in focal PVL. But, in many cases, WMI is widespread and incorporates periventricular, subcortical, and callosal white matter, as well as the internal capsule. WMI, and in particular PVL, has two distinct histopathological appearances, described as either cystic or non-cystic (diffuse) WMI. Cystic WMI typically affects all types of cells and is therefore considered the more severe type and is closely linked with CP, whereas diffuse PVL mainly targets pre-oligodendrocytes and is considered less severe but nonetheless is linked to cognitive and behavioral impairments, and CP (9). It is generally considered that the gray matter is not as susceptible to preterm insults as is white matter, but the pre-oligodendrocytes also present in gray matter are not spared, leading to damage involving the cerebral cortex, thalamus, and basal ganglia (26, 27).

There is a growing understanding of the etiology of preterm brain injury, likely involving one or more interactions between fundamental immaturity of the brain, vulnerability of white matter developmental processes, and the adverse effects of two principal upstream insults: hypoxia–ischemia and infection/inflammation. Hypoxia–ischemia and infection/inflammation are relatively common in the preterm period, and have profound adverse effects on white matter development (28–30).

## Hypoxia–Ischemia and White Matter Injury

After an hypoxic-ischemic insult, microglia and macrophages within the brain’s white matter exhibit immunoreactivity for interleukin-6 (IL-6) and tumor necrosis factor-α (TNF-α), and infiltrate to lesion sites. Astrocytes become hypertrophied and diffuse gliosis is evident within 24 h. Loss of oligodendroglial lineage cells and impairment of myelinogenesis is evident within 10 days following hypoxia–ischemia (9, 31–35). When the insult has been prolonged or severe, brain injury is exacerbated through influx of cytokines and chemokines via the damaged blood–brain-barrier (BBB), thereby further increasing inflammatory mediators within the brain (36).

In response to a significant hypoxic-ischemic insult, secondary pathways of injury are also initiated and evolve over days. These adverse pathways include mitochondrial dysfunction, excitotoxicity, apoptosis, oxidative stress, and initiation of additional inflammatory processes (37). A further adverse effect of hypoxia–ischemia is the disruption of normal growth and differentiation factors driving brain development, decreasing concentrations of signaling proteins and nutrients that include neurotrophic factors vital for inhibition of programed cell death (38, 39). For example, brain-derived neurotrophic factor (BDNF), neurotrophin-3 (NT-3), and NT-4/5 play important roles in promoting neuronal growth and differentiation, connective plasticity, and neuronal survival through their interaction with tyrosine kinase β-receptors, but these are each affected in the preterm brain in response to hypoxia–ischemia (40). Additional neuron and glial cell loss occurs over days and weeks after a sentinel insult, resulting from chronic deprivation of neurotrophic factors, decreased synaptic input from neighboring cells, and loss or recruitment failure of local neural and glial stem and progenitor cells (41, 42). The severity and duration of neurotrophic factor deprivation directly correlates to long-term neurological outcome (38, 42).

In addition, activated astrocytes and microglia mediate the release of reactive oxygen species (ROS) and reactive nitrogen species (RNS), leading to increased protein nitration and oxidative stress in response to hypoxia–ischemia and brain inflammation (43). In the context of preterm birth, a compromised intrauterine environment may induce excess release of free radicals and, combined with the transition to an extra-uterine high oxygen environment, may overwhelm endogenous antioxidant enzymes, resulting in preferential death of pre-oligodendrocytes and contributing to the development of WMI within the preterm brain (43–46).

## Infection/Inflammation and White Matter Injury

Fetal and neonatal exposure to infection and/or inflammation is recognized as a principal contributor to preterm birth and WMI. Maternal intrauterine infection including chorioamnionitis is associated with increased levels of pro-inflammatory cytokines (IL-6, IL-8, TNF-α, and IL-1b) in the amniotic fluid and cord blood (47–49) and is one of the most important causes of preterm birth <30 weeks of gestation (50). Maternal intrauterine infection presents a significant risk for WMI and CP (51, 52). Neonatal sepsis is also a risk factor for WMI in infants that were born preterm (53, 54).

Adverse inflammatory stimuli during fetal or neonatal life induce a systemic and central nervous system (CNS) response via activation of innate and adaptive immune systems. Microglia are the primary mediators of the brain’s immune response, mediating the pro- and anti-inflammatory response to remove pathogens, via binding of toll-like receptors (TLRs) with ligands, pathogen-associated molecular patterns (PAMPs), and/or danger-associated molecular patterns (DAMPs) (55, 56). However, prolonged microglial activation can cause brain injury (55). Microglia are at their peak density in white matter during the WMI-vulnerable period (57), making them fundamental in producing WMI (44). Lipopolysaccharide (LPS), an endotoxin of gram-negative bacteria and a form of PAMP, and binds receptors including TLR-4 and CD14 on microglia, initiating a signal transduction cascade that ultimately activates transcription factors such as nuclear factor-kappaB (NF-κB). In turn, this leads to up-regulation of cytokines, chemokines, and complement proteins, and over time this response can sensitize the developing brain to secondary insults thereby contributing to sustained CNS inflammation. Cytokines may directly act upon oligodendrocytes to induce cell death, as evidenced by *in vitro* studies on human oligodendrocytes where TNF-α and interferon-γ (IFN-γ) induced dose-dependent cell necrosis (58). *In vivo* administration of high-dose LPS to preterm fetal sheep results in significant cerebral hemodynamic changes that cause cerebral ischemia and PVL-like fetal brain injury (59), while low-dose LPS, insufficient to cause fetal hypoxia, induces diffuse WMI and microglial invasion, where the degree of microglial activation is correlated to the presence of WMI (60).

The downstream pathways that result from hypoxia–ischemia, or inflammatory stimuli, are complex and are not mutually exclusive. Hypoxia–ischemia and increased ROS are known to induce an inflammatory reaction and, conversely, pro-inflammatory mediators lead to the generation of free radicals and oxidative stress. This interaction is driven by NF-κB. In normal-state resting cells, the NF-κB protein complex remains within the cytoplasm, bound to inhibitory IκB protein. Pro-inflammatory cytokines, LPS and viruses cause proteolysis of IκB, allowing dissociation from NF-κB, and the nuclear translocation of NF-κB where it activates gene transcription (61). Additionally, tissue hypoxia and oxidative stress can modulate NF-κB release (61). Thus, hypoxia–ischemia can induce inflammation via microglial activation, and conversely infection/inflammation can induce hypoxia–ischemia through hypotension (62). Indeed, preterm infants have been shown to have higher risk of WMI when chorioamnionitis and placental perfusion deficits are present together (63).

## Limitations of Current Treatments of White Matter Injury

Although there have been major clinical and scientific advances in neonatal care over the last decade, currently only antenatal corticosteroid are proven to reduce the risk of intraventricular hemorrhage (IVH) (64). Other strategies in preterm infants, such as use of erythropoietin, melatonin, indomethacin, antenatal magnesium sulfate, therapeutic hypothermia, or delayed cord clamping remain at the experimental investigation stage and are not of proven benefit (65, 66). Thus, current management for preterm brain injury has, until now, been restricted to supportive strategies.

One of the biggest hurdles for identifying neuroprotective strategies for preterm infants is the multi-faceted etiology of the brain damage. As described in the section above, the primary antenatal causal factors that may induce brain injury include maternal/fetal infection and/or chronic placental perfusion insufficiency (67, 68). Postnatal factors may exacerbate or cause brain injury, including repetitive subacute/chronic hypoxia–ischemia due to poor lung function and ventilation, and free radical imbalance following oxygen reperfusion in response to a high oxygen extra-uterine environment or oxygen administration (44, 46, 69). Neonatal chronic cerebral hypoperfusion, hypotension, hypocarbia, or symptomatic persistent ductus arteriosus (70, 71), IVH with or without post-hemorrhagic ischemia or hydrocephalus (72), infection (53, 54), hypoglycemia, and glucocorticoid administration are also involved in the progression of brain injury (68, 73). Moreover, even preterm birth without exacerbating factors can result in subtle white matter pathology (69). Thus, it can be appreciated that unlike term neonatal HIE, these insults do not necessarily occur around the time of delivery, and it may therefore be difficult to recognize the timing of the onset of a sentinel (or exacerbating) insult. It would therefore be likely that preterm brain injury would be best treated with a therapy, or therapies, with multiple neuroprotective mechanisms and with a long therapeutic window. Any therapy should be targeted at WMI as the predominant neuropathology. Additionally, such a treatment would be aimed at one or more of the following – reducing inflammation and free radical attack, halting the progression of cell death programing, and/or replacing damaged oligodendrocytes in order to remodel areas of WMI and normalize myelination. We will present data to support the therapeutic potential of neonatally administered UCB-derived cells, for protection and repair of the preterm brain.

## Stem Cells

Stem cells are characterized by their ability to undergo self-renewal and to differentiate into multiple cell types. In general, stem cells can be classified into three major categories on the basis of their source, namely embryonic stem cells (ESCs), fetal-derived stem cells, and adult stem cells. ESCs are pluripotent, are able to generate cells from all three germ layers and can be maintained in culture indefinitely (74), providing a limitless source of precursor cells for the regeneration of damaged tissue. However, due to the pluripotent nature of ESCs they are also tumorigenic and transplantation of these cells currently presents significant safety concerns (75), and has cautioned their use in clinical trials. ESCs are also obtained from embyros, presenting ethical issues. With recent advances, it has become possible to reprogram somatic cells and generate induced pluripotent stem (iPS) cells (76). These cells may be able to overcome some of the limitations of ESCs, i.e., ethical issues, and enable the generation of patient-specific iPS cell lines. However, they are also tumorigenic (77), and this issue remains unresolved. Adult stem cells, or stem-like cells, include mesenchymal stromal cells (MSCs), that can be obtained from a number of sources including bone marrow, adipose tissue, and dental pulp, and neural progenitor cells (NPCs) that are found in the subventricular zone of the brain and comprise multipotent stem/progenitor cells that can differentiate down the neural lineage, including to neurons and glial cells (78). NPCs can be isolated from the adult brain and expanded for several passages whilst retaining their undifferentiated state. Lastly, fetal-derived stem-like cells can be obtained from placental tissue (79) and UCB (80) and include placental and umbilical cord MSCs (UC-MSCs), amnion epithelial cells (AECs), HSCs, and endothelial progenitor cells (EPCs) (81). Given these cells are isolated from fetal tissue, they tend toward greater differentiation and expansion potential than adult stem cells. Fetal-derived stem cells can be easily isolated from tissue that is routinely discarded at birth, they are abundant due to the large number of births each year, and their collection raises no ethical concerns.

Stem-like cells sourced from placenta and umbilical cord (UC) have been studied pre-clinically for treatment of a variety of diseases including multiple sclerosis (82, 83), stroke (84, 85), bronchopulmonary dysplasia (86, 87), and CP (88, 89) and may be beneficial for reducing disease burden in these conditions. For MSCs alone, there are currently >400 clinical trials listed on clinical trials.gov (search: “mesenchymal stem cell”). However, there are numerous clinics around the world that are already capitalizing on the *promise* of stem cell treatment and are offering stem cell therapies for financial gain to families of those with conditions including CP. This has been coined stem cell tourism (90). It is therefore imperative that well-planned and controlled pre-clinical and clinical trials are conducted to establish the safety, short-, and long-term efficacy, and mechanisms by which stem cell therapies may provide benefit, which in turn will enable treating clinicians and patients to make informed decisions regarding the use of stem cell treatments.

## Umbilical Cord Blood

Umbilical cord blood is a rich source of HSCs, accounting for 0.5–1.0% of mononuclear cells (MNCs) in term UCB (91), used to treat patients with abnormal hematopoietic conditions, childhood leukemia, or metabolic diseases (92). HSCs are positive for CD34 and CD45, and defined by their capacity to self-renew and give rise to multiple blood lineages. Traditionally, bone marrow-derived HSCs were used to treat these conditions, however, UCB is easier to obtain, less expensive and less likely to trigger a deleterious immune response or rejection in the recipient (93). HSCs from human UCB (hUCB) are also more primitive than bone marrow-derived HSCs, have longer telomeres, have a higher colony-forming capacity and can repopulate blood lineages over a long period of time (94, 95). Given these advantages, more than 3000 hUCB transplants are now performed each year for blood and other disorders (96). Other strong advantages for the use of UCB for transplants include that it can be tissue typed, screened for viral biomarkers, processed and banked, allowing the supply for both urgent and directed transplants (97) and the volume and number of cells that can be attained is generally very good. In term births, a large volume of UCB can be collected [38–42 weeks: 102 ± 30 ml, containing 11.3 ± 6.2 × 10^8^ total nucleated cell (TNC)] (91). However, in preterm birth, or in pregnancy complications [such as intrauterine growth restriction (IUGR)], there is reduced UCB volume for collection [34–37 weeks: 90 ± 32 ml, 7.7 ± 4.8 × 10^8^ TNC; 25–33 weeks: 62 ± 31 ml, 3.3 ± 3.5 TNC] (91), which is problematic if UCB collection is required or requested. Furthermore, it is not known how antenatal complications, such as IUGR or chorioamnionitis, may change the composition of the stem and stem-like cells present in the UCB, and whether differences in cell composition may impact its therapeutic utility.

Umbilical cord blood is not only a useful source of HSCs, but also contains a number of other stem/progenitor cell types including MSCs and EPCs (80). Moreover, UCB is a rich source of immunosupressive cells, such as regulatory T cells (Tregs) (98). MSCs are multipotent adult progenitor cells that have a broad potential for repair of injured tissue. MSCs are characterized by their morphology, phenotype, and differentiation potential to form osteoblasts, chondrocytes, and adipocytes (82). MSCs are a plastic adherent cell population with the absence of CD34, CD45, and CD133, and are positive for CD13, CD29, CD44, CD73, and CD90 (80, 99). MSCs can indeed be isolated from UCB, but at a very low frequency and cellular fraction, with success rates for isolation ranging from 40 to 60% (99, 100) and, in one study, only 8% of UCB units could be effectively expanded into MSC-like colonies (100). While the frequency of MSCs is low in UCB (0.002% of MNCs in term UCB) (101), UCB–MSCs show a strong proliferation capacity and can be maintained longer in culture than MSCs derived from other sources (99). Furthermore, following exposure to neural differentiation factors, hUCB–MSCs express a number of neural cell antigens, including glial fibrillary acidic protein (found in astrocytes), TuJ-1 (neural progenitor), vimentin, and nestin (102).

Endothelial progenitor cells, isolated from bone marrow, peripheral blood, and UCB can be differentiated into mature endothelial cells *in vitro* and, in animal models of ischemia, can incorporate into sites of active angiogenesis to stabilize and promote the growth of new blood vessels (103). While EPC classification remains contentious, they are generally characterized by the expression of CD133, CD34, and vascular endothelial growth factor (VEGF) receptor-2 (104). EPCs are estimated to make up 1–2% of HSC-containing CD34+ cell fraction in term UCB, representing 1 in 10^7^ MNCs (105). Despite the low number, EPCs isolated from hUCB have been shown to have a stable endothelial phenotype and a higher proliferative capacity compared to those isolated from peripheral blood, making UCB a superior source for the isolation of EPCs. Studies are now being conducted to optimize the isolation of the three major cell types (EPCs, HSCs, and MSCs) from single UCB units (80).

Regulatory T cells should also be considered as potential useful cells to be isolated from UCB. Tregs are immunosupressive T cells that can maintain self-tolerance, prevent autoimmunity, inhibit rejection of transplants, and regulate the immune response to infectious disease (106). Tregs isolated from UCB exhibit a predominantly naïve phenotype, which is associated with a significantly enhanced proliferative potential compared to adult Tregs (107). It has been suggested that the low incidence of graft-versus-host-disease (GVHD) associated with UCB transplants is due to the presence of Tregs (108), adding to their importance for UCB transplants and their potential utility for treatment of inflammatory conditions.

Optimal selection of UCB units for HSC transplants includes determination of TNC content, CD34+ cell count, and HLA and blood group matching of the recipient and donor (97). However, there is sparse information related to other cells of interest within UCB. Given the increasing likelihood that children born preterm may request autologous UCB cell collection and treatment for brain injury (see below), it is imperative that we investigate the similarities and differences between term and preterm UCB. This knowledge will inform the design of clinical trials that will decide whether autologous or allogeneic UCB transplants will be best placed to treat neurological impairments in preterm-born infants. To date, it has been shown that the frequency of CD34+ cells in preterm neonates was twofold increased compared to those in term neonates (109) and, for a given gestational age, each 500 g increase in birth weight contributed to a 28% increase in CD34+ cell counts (110). In preterm infants, the immunophenotypic profile of UCB–CD34+ cells shows a significantly higher expression of CD33, and a lower expression of CD38, CD117, and HLA-DR, indicating preterm UCB has a higher percentage of primitive CD34+ subsets, while term UCB has a higher percentage of committed cells (111, 112). With specific regard to EPCs, preterm (28–34 weeks) UCB units have a fourfold increase in endothelial colony-forming cells compared to term UCB (113). However in compromised placental conditions such as preeclampsia, EPCs are decreased in term UCB, and not different to those in preterm UCB (114). Similar to other cell types, MSC population is also richer in preterm UCB compared with term, with a significant inverse correlation between the gestational age and presence of MSCs (101, 115). Furthermore, studies to date have predominantly assessed cell number and not cell function over gestation, where functionality may be a more important marker of efficacy than absolute cell number. As has been shown with hAECs, while preterm cells have a high proliferative capacity, they are functionally immature and cannot differentiate into other cell types (116). The presence of Tregs has also been assessed over gestation, and is reportedly increased in preterm UCB compared to term UCB (107), but Tregs from preterm UCB secrete significantly less IFN-γ (117). Furthermore, Tregs in UCB from IUGR infants at term were decreased compared to those in UCB units from appropriately grown babies (118).

Most studies to date examining perinatal brain injury have utilized UCB–MNCs (Table 1), but UCB–MNCs is composed of a variety of cells of interest including immature T cells, B-cells, monocytes, and stem-like cells including HSCs, EPCs, and MSCs. The fraction or combination of UCB cells responsible for neural repair remains to be established. UCB–MSCs have attracted interest for some time because of their multilineage differentiation potential, strong capacity for immune modulation, and low immunogenicity. Indeed, expanded hUCB–MSC transplantation has shown promise in protecting against perinatal brain injury in pre-clinical animal studies (119–121). Despite this, the clinical application of purified UCB–MSCs is currently limited by their low numbers and low success rate for isolation. On the other hand, CD34+ cells have been shown to reduce brain injury in neonatal hypoxic-ischemic mice, with a transient augmentation of cerebral blood flow in the peri-infarct area (122). UCB–CD133+ cells, the fraction enriched for EPCs and HSCs, also reduces infarct volume in a rat model of stroke (123).

**Table 1:** **Outcome of umbilical cord blood interventions in neonatal hypoxia–ischemia**.

Cell type	Animal model	Administration			Engraftment		Histology assessments		Functional assessments		Other	Reference
	Injury type	Timing	Dose	Route	Days	Results	Days	Outcomes	Days	Outcomes	
hUCB–MNCs	P7 rats, HI 80 min	24 h after HI	1 × 10^7^ cells	IP	21 days	Many cells in ischemic hemisphere. No sign of transdifferentiation	NA	NA	21 days	Alleviation of spastic paresis		Meier 2006 (127)
	P7 rats, HI 120 min	24 h after HI	1 × 10^7^ cells	IV jugular	21 days	Few cells in brain tissue	21 days	No change in volume of injured hemisphere	21 days	No change on spatial memory deficit		de Paula 2009 (192)
	P7 rats, HI 90 min	3 h after HI	2 × 10^6^ cells	IP	2 days	Few cells in ischemic cortex and striatum	2 days	Decreased neuronal death in striatum, and microglial activation in cortex	4, 7 days	Improved developmental sensorimotor reflexes only at 4 days		Pimentel-Coelho 2010 (134)
	P7 rats, HI 150min	2–3 h after HI	1.5 × 10^4^ cells (±mannitol)	IV jugular	14 days	Few cells in ischemic hippocampus	NA	NA	7, 14 days	20–25% improvement in rotarod and elevated body swing tests	Increased growth factors in brain, CA1 dendrites	Yasuhara 2010 (138)
	P7 rats, HI 80 min	24 h after HI	1 × 10^7^ cells	IP	42 days	Many cells in peri-infarct area	42 days	No change in size of hemispheric lesion	42 days	Improved sensorimotor function, cortical maps, and receptive fields, and reduced hyperexcitability		Geissler 2011 (135)
	P7 rats, HI 80 min	24 h after HI	1 × 10^7^ cells	IT	14 days	hUCB cells were localized in astrocyte-rich zone	2, 14, and 44 days	Decreased activation of microglia/macrophages and reactive astrogliosis, and reduced peri-lesional astrocytic wall	14, 44 days	Improved motor function (forelimb use bias, muscle strength and distal spasticity) both short- and long-term	Downregulation of Connexin 43	Wasielewski 2012 (136)
	P7 rats, HI 80 min	24 h after HI	1 × 10^7^ cells	IP	NA	NA	2, 14 days	Decreased lesion-induced apoptosis, increased neurons	NA	NA	Increased the expression of proteins Tie-2, occludin, BDNF and VEGF in the lesioned brain	Rosenkranz 2012 (141)
	P7 rats, HI 120 min	2 h after HI	1 × 10^6^, 1 × 10^7^, 1 × 10^8^ cells	IV jugular	7 days	Cells in the cortex and the hippocampus	8 weeks	No change in low-dose group. Decreased brain atrophy in medium- and high-dose groups	8 weeks	Cognitive improvement at the highest dose only		de Paula 2012 (133)
	P7 rats, HI 90 min	24 h after HI	1 × 10^7^ cells	IV jugular	1, 3, and 10 weeks	Many cells were in ischemic periventricular region at 1 week, but very few at 3 and 10 weeks	10 weeks	No decrease in tissue loss volume, decreased neuronal loss in neocortex	10 weeks	Improved performance in a battery of behavioral tests		Bae 2012 (139)
	P7 rats, HI 120 min (+cyclosporin A)	24 h after HI	3 × 10^6^ cells	IVen	NA	NA	24, 72 h, 7, 14 days	Decreased neuronal loss in cortex and CA1 of the hippocampus	NA	NA	Increased Shh and Gli1 protein levels	Wang 2014 (137)
hUCB–CD34+	P12 SCID mice, MCAO	48 h after HI	1×10^5^ cells	IV femoral	24 h, 10 days	Few cells at 24 h, very few at 10 days	7 weeks	Decreased brain atrophy	9 days, 7 weeks	No effect on rotarod or open-field tests	Transient augmentation of CBF in peri-infarct area	Tsuji 2014 (122)
hUCB–MSCs (passage 10)	P7 rats, HI 150 min (+cyclosporin A)	3 days after HI	1 × 10^5^ cells	Intra-cerebral	7 days	Differentiation into astrocytes but not neurons	28 days	Reduced cortical neuronal loss	14, 21, 28 days	Improved neurological score		Xia 2010 (119)
(passage 5)	P10 rats, MCAO	6 h after HI	1 × 10^5^ cells	IVen	NA	NA	28 days	Decreased apoptosis, microglial activation and astrogliosis in penumbra	28 days	Functional improvements (rotarod and cylinder test). GM1 enhanced the behavioral recovery	Improved survival and body weight gain, decreased lesion volume on MRI	Kim 2012 (120)
hUC-MSCs (passage 3)	P7 rats, HI 120 min (+cytarabine)	24 or 72 h after HI	5 × 10^6^ cells (±GM1)	IP or IV jugular	35 days	More cells were in ischemic frontal cortex after iv than ip, with neural differentiation around infarct region	35 days	Decreased gliosis in ischemic regions	7 days, 20 days, 4 weeks	More improved locomotor function in animals given cells at 24 h than 72 h		Zhang 2014 (130)

*BDNF, brain-derived neurotrophic factor; CA1, cornu ammonis 1 of the hippocampus; CBF, cerebral blood flow; GM1, ganglioside; HI, hypoxic–ischemia (unilateral ligatiion of the carotid artery followed by 8% oxygen systemic hypoxia); hUCB–MNCs, human umbilical cord-mononuclear cells; IP, intraperitoneal; IT, intrathecal; IV, intravenous; IVen, intraventricular; MCAO, middle cerebral artery occlusion; MSCs, mesenchymal cells; NA, not applicable; P, postnatal day; UC, umbilical cord; VEGF, vascular endothelial growth factor*.

In addition to stem-like cells, other cellular fractions in UCB have also been shown to have potentially important neuroprotective roles. When hUCB–MNCs was depleted for CD14+ monocytes, there was no decrease observed in microglial activation or functional recovery following administration (124), suggesting that monocytes are essential for mediating the neuroprotective benefits of hUCB cells in hypoxic-ischemic rats. In addition, a further study showed that a single injection of hUCB-derived T cells (CD4+) induced endogenous NPC proliferation for 2 weeks and promoted increased neuronal cell survival in rats (125). The therapeutic effects of stem cells are now thought to be independent of tissue engraftment (89, 126–129), although many studies have shown that transplanted UCB cells can migrate selectively toward ischemic areas of damaged brain (127, 130). It is widely considered that regenerative effects of stem cells are principally derived from indirect paracrine and trophic effects, and increasing the regenerative capacity of the brain, rather than via direct cell replacement (38, 128, 129, 131, 132). However, it is important to note that the studies referred to above have utilized hUCB in a xenogeneic setting. As such, the ability of the transplanted cells to survive and differentiate may be compromised (133). To our knowledge, the ability of autologous UCB cells to home to the site of injury and differentiate into neurons or neuroglial cells has not been investigated. Thus, the engraftment potential of UCB cells remains poorly characterized, and requires further investigation in studies using autologous transplantation.

## Neuroprotective Properties of Umbilical Cord Blood

A number of studies have demonstrated significant and reproducible neuroprotective effects in rodent models of term neonatal hypoxia–ischemia using UCB–MNCs (127, 133–139), UCB–MSCs (119, 120), or UCB–CD34+ cells (122). Meier and colleagues first showed that intra-peritoneal administration of hUCB–MNCs alleviated spastic paresis in the Rice–Vannucci model of neonatal hypoxic-ischemic rats (127, 140). Following this, other rodent studies have shown that hUCB–MNCs induce significant improvements in sensorimotor performance (134–136, 138) and reduction in neuronal loss (133, 134, 137–139, 141). Recent studies also showed long-lasting neuroprotective effects of hUCB–MNCs in behavioral and cognitive outcomes at 8 and 10 weeks after ischemic insult (133, 139), with decreased brain atrophy (133). Animals treated with intra-cerebral hUCB–MSCs also demonstrated improved neurological function and tissue repair (119, 120).

From pre-clinical results obtained to date, we hypothesize that UCB cells may act in a neuroprotective manner via diverse actions, including anti-inflammatory effects, immunomodulation, and neurotrophic growth factor release to promote endogenous neurogenesis.

## Anti-Inflammatory and Immuno-Modulatory Actions of Umbilical Cord Blood

A principal mechanism whereby UCB cells regulate neurological repair is via anti-inflammatory actions. UCB administration can dampen the expression of pro-inflammatory cytokines (IL-1α, IL-6, IL-1β, and TNF-α), enhance anti-inflammatory cytokines (IL-10), secrete chemotactic proteins (monocyte chemotactic protein 1), and modulate immune macrophage and T cell function (142, 143). As described above, hypoxia–ischemia induces an acute brain inflammatory response with activation of microglia and macrophages and reactive astrogliosis associated with peri-lesional up-regulation of connexin 43, the major astrocytic gap junction protein (144). Administration of hUCB–MNCs normalizes inflammatory balance, reduces microgliosis and astrogliosis (134, 136), and down-regulates connexin 43, which in turn restores BBB function to moderate inflammatory cell influx into the brain (136).

## Neurotrophic Factor Actions of Umbilical Cord Blood

Transplanted UCB–MNCs or MSCs reportedly enhance neurological recovery via secretion of a wide variety of trophic factors including BDNF, glial cell line-derived neurotrophic factor (GDNF), nerve growth factors NT-3 and NT-5, angiogenin, VEGF, fibroblast growth factor-2, and epidermal growth factor. Together, these act to promote endogenous neuronal growth and neurogenesis, angiogenesis, encourage remyelination, and synaptic connections, and decrease cellular apoptosis (141, 145–147). Transplantation of hUCB is associated with reduced levels of cleaved-caspase-3 protein in hypoxic-ischemic newborn rats, indicative of reduced apoptosis, with BDNF identified as playing a role in inhibition of apoptosis and inflammation (141). Further, hUCB cell administration after hypoxia–ischemia increases expression of Tie-2 and occludin proteins, and increases expression of VEGF, indicating that UCB transplantation may increase endogenous angiogenesis and improved BBB integrity within the damaged brain. *In vivo*, MSCs provide trophic neuroprotection following injury by secreting physical tissue scaffold to surrounding tissues, while UCB–CD34+ cell transplantation enhances functional recovery and reduces both infarction and apoptosis in a rat model of spinal cord injury, mediated by the production of both VEGF and GDNF (148). UCB-derived CD133+ cells promote a threefold improvement in axonal regrowth, a 35% reduction in apoptosis and vascular and neuronal protection following hypoxia on organ co-culture of brain motor cortex cells and spinal cord from postnatal day 3 rats, suggesting that the trophic effects from CD133+ cells contributes to neuroprotection (149).

Hypoxia–ischemia stimulates endogenous proliferation of NPCs (150). However, if an insult is severe, brain damage still occurs, and the restorative proliferation of NPCs may be ameliorated, with activated NPCs failing to survive to mature neurons (150, 151) or differentiating into astrocytes (152). Recently UCB–MNC transplantation has been shown to promote the proliferation of endogenous NPCs, and reduce glial differentiation, an action mediated via the Sonic Hedgehog signaling pathway, resulting in the alleviation of brain injury in hypoxic-ischemic neonatal rats (137). These results support the ability of UCB cells to respond to insult with paracrine and trophic actions, initiating a regenerative environment mediated by resident cell populations within the brain.

## Preterm Brain Injury Animal Models and Cell Therapy

The majority of experimental studies described above that have investigated the neuroprotective actions of hUCB have been undertaken in rodent models of neonatal (term) hypoxic-ischemic brain injury (119, 120, 122, 127, 133–139). Injury to the human brain at the time of term birth induced by hypoxia–ischemia predominantly causes deep gray matter neuronal injury within the basal ganglia and hippocampus, together with injury to neighboring white matter – this is appropriately reflected in rodent and large animal studies of term hypoxia–ischemia. However, this distribution of injury is quite different in preterm WMI, reflecting susceptibility and region-specific effects following hypoxia–ischemia and other insults as brain maturation progresses.

Animal models exploring the injury profile and mechanisms of preterm pre-oligodendrocyte and WMI, have utilized either an hypoxic-ischemic insult, or exposure to LPS-induced inflammation. Excitotoxicity models such as administration of excitatory amino acid agonists quinolinic acid and ibotenate have also been used (26, 153). It is important to note that preterm infants mostly suffer hypoxic-ischemic insults that are subacute or chronic, in contrast to term infants where HIE is principally due to an acute severe insult (154). Irrespective of which experimental insult is utilized, the maturational age of the CNS is critical (26). In addition, the choice of species is important. It is well described that induction of predominant WMI is problematic in rats and mice due to the different CNS anatomy of rodents that, besides being non-gyrencephalic and having a different vascular anatomy, demonstrates a much lower white/gray matter ratio than in humans. In contrast, the pattern of WMI in rabbits, cats, dogs, and sheep has a distribution and morphological appearance closer to that of human preterm brain injury induced by either hypoxia–ischemia or LPS administration (26, 59). The fetal rabbit brain myelinates with a similar perinatal time course to the human, and maturation of oligodendrocytes begins antenatally (155). *In utero*, hypoxia–ischemia to the preterm rabbit fetus causes postnatal hypertonic motor deficits that resembles CP, making rabbits a very good model for postnatal behavioral studies (156). Many further studies have been undertaken utilizing fetal sheep models of preterm WMI because of their abundance of cerebral white matter, their anatomic similarities to the preterm infant, and an ability to monitor the systemic and brain response to insult (37, 59, 157). However, because of cost, availability of antibodies/sequence data and genetically modified animals, rodent WMI studies are valued as complementary models (26). To date, only hAECs have been examined in a non-rodent (fetal sheep) model of preterm WMI. Yawno and colleagues demonstrated that administration of hAECs suppressed the up-regulation of activated microglia, and reduced gray and WMI in response to LPS in preterm fetal sheep (88) (Table 2).

**Table 2:** **Outcome of cell-based interventions in preterm brain injury**.

Cell type	Animal model	Administration			Engraftment		Histology assessments		Functional assessments		Others	Reference
	Injury type	Timing	Dose	Route	Days	Results	Days	Outcomes	Days	Outcomes	
hUCB–MNCs	P2 rats, MCAO	48 h after stroke	1 × 10^6^ cells	IV	NA	NA	4 days	Reduced white matter damage	NA	NA	UCB–MNCs directly reduced apoptosis of oligodendrocytes cultured under oxygen glucose deprivation *in vitro*	Hall 2008 (158)
hUCB–MNCs	P5 rats, excitotoxicity (ibotenate)	within 6 h or 24 h after injection	1 × 10^6^ or 10^7^ cells	IP or IV	5 days	No cells detected	5 days	No changes in lesion size, microglial activation, astrogliosis, or cell proliferation. Increased white matter damage with increased microglial activation by ip administration	NA	NA		Dalous 2013 (89)
hUC-MSCs (Passage 3)	P3 rats, HI 240 min	0, 1, 2 days after HI, once a day	1 × 10^6^ cells, 3 times	IP	24 h	Cells migrated mainly toward the injured hemisphere	7, 18 days	Increased mature oligodendrocytes counts. Decreased astrocytosis and microglial activation	27 days	Improved exploratory behavior, mental stress and motor function		Zhu 2014 (174)
hUCB–MSCs	P4 rats, blood injection into lateral ventricle	P6	1 × 10^5^ cells	IVen	NA	NA	28 days	Improvements of corpus callosal thickness and myelin basic protein expression reduction. Attenuation of astrogliosis and cell death	28 days	Improved behavioral tests (negative, geotaxis test and rotarod test)	Attenuation of post-hemorrhagic hydrocephalus development by MRI. Decreased inflammatory cytokines expression in CSF (IL-1α, IL-1β, IL-6, and TNF-α)	Ahn 2013 (121)
hAECs	117 days GA fetal sheep, LPS	0, 6 and 12 h after LPS	IT 1.8 × 10^8^ cells, or IV 9 × 10^7^ cells, or IT 9 × 10^7^ + IV 9 × 10^7^ cells	7d	Cells were detected in 2 of 14 fetal brains	7 days	Decreased activated microglia in the cortex, subcortical and periventricular white matter. Decreased apoptosis in the cortex and periventricular white matter	NA				Yawno 2013 (88)

*CSF, cerebral spinal fluid; GA, gestational age; HI, hypoxic–ischemia (unilateral ligatiion of the carotid artery followed by 6% oxygen systemic hypoxia); hAECs, human amnion epithelial cells; hUCB–MNCs, human umbilical cord-mononuclear cells; IP, intraperitoneal; IT, intrathecal; IV, intravenous; IVen, intraventricular; MCAO, middle cerebral artery occlusion; MSCs, mesenchymal cells; MRI, magnetic resonance imaging; NA, not applicable; P, postnatal day; UC, umbilical cord; IL, interleukin; LPS, lipopolysaccharide; TNF-α, tumor necrosis factor-α*.

## Preterm Brain Injury and Umbilical Cord Blood

Hall and colleagues demonstrated that in postnatal day 2 rats, intravenous hUCB–MNC administration preserves white matter structures following an hypoxic-ischemic insult. This timeframe corresponds to the period of white matter vulnerability in human preterm infants between 24 and 30 weeks of gestational age (158). Specifically, IV infusion of hUCB–MNCs at 48 h post-ischemia reduced WMI based on quantification of myelin basic protein. A direct protective effect of UCB–MNCs on oligodendrocyte injury induced by oxygen/glucose deprivation (OGD), which produces hypoxic-ischemic-like injury *in vitro*, was also identified (158). Although the data are limited, it appears that hUCB–MNCs have therapeutic potential for the protection of oligodendrocytes and thereby prevention of WMI in a premature rat model of ischemia.

## Antiepileptic Effects of Umbilical Cord Blood

The incidence of seizures in very low birth weight infants is 5.6%, while the occurrence in those infants identified as having PVL is 18.7% (159, 160). Seizures are typically observed in more severe cases of PVL and those born at lower gestational ages and birth weights (159, 160). Recent studies demonstrate the antiepileptic actions of hUCB–MNCs. Transplantation of hUCB–MNCs 90 min after the onset of status epilepticus in rats, induced by lithium and pilocarpine chloride, protected against neuronal loss in the hippocampus for up to 300 days. Additionally, MNC-transplanted rats had reduced frequency and duration of recurrent seizures, suggesting early administration could protect against the establishment of epilepsy (161). Furthermore in a single case of an infant with infantile spasms (West syndrome) and X-linked T/B + NK-severe combined immunodeficiency, allogeneic UCB transplantation together with topiramate and immune-modulating agents (corticosteroids, intravenous immunoglobulin, and tacrolimus) improved seizures, possibly contributed by an immuno-modulatory effect of UCB–MNCs (162).

## Suppression of Excitotoxicity by Umbilical Cord Blood

Suppression of excitotoxicity is a further important subject of investigation for protecting the developing brain. The potential therapeutic effects of stem cells in animal models of excitotoxic brain injury have been examined using the *N*-methyl-d-aspartate receptor agonist, quinolinic acid, to induce apoptosis and cleaved caspase-3 and excitotoxic damage in the neocortex, hippocampus, striatum, white matter, and subventricular zone, in the newborn mouse brain. Injection of human embryonic germ cell-derived NPCs partially restores the complement of striatal neurons, with engraftment of the transplanted cells in injured sites and their differentiation into neuronal and glial cells (163). In contrast, intra-peritoneal and intravenous hUCB–MNCs administration could not promote brain repair in ibotenate-induced excitotoxic brain lesions in neonatal rats. The authors of this recent study did, however, suggest that the intra-peritoneal injection of high amounts of hUCB–MNCs may have aggravated WMI, possibly due to systemic inflammation (89).

## Antioxidant Effects of Umbilical Cord Blood

There is increasing evidence that stem cells, especially young cells, possess antioxidant potential, which may then contribute to anti-apoptotic effects (164–166). A recent paper showed that hUCB–NPCs, a neuronal phenotype differentiated from collagen-adherent hUCB–MNCs, induced neuroprotection via an antioxidant effect, decreasing free radical levels by 95% (167). Human MSCs *in vitro* also scavenge oxygen and nitrogen free radicals, constitutively express antioxidant enzymes, and themselves are highly resistant to oxidative stress-induced death (166). It is still unclear whether cells derived from UCB can mediate tissue oxidative stress *in vivo*. Since oxidative stress is known to play an important role in the progression of brain injury in preterm infants, this is a crucial consideration for the ability of UCB–MNCs to mediate the progression of preterm brain injury.

## Vascular Development, Intraventricular Hemorrhage, and Umbilical Cord Blood

Preterm infants are highly vulnerable to IVH due to their maturation-dependent vascular vulnerability, localized to the area of the periventricular germinal matrix and possibly in part due to a coagulation system deficit of prematurity (9, 168). Preterm infants predominantly develop IVH in the first week after birth (169). While grades 1 and 2 IVH cause little neurological harm, >50% of infants with severe IVH (grades 3 and 4) die or develop post-hemorrhagic hydrocephalus (PHH). The incidence of severe IVH in very preterm infants ranges from <5 to 20% (170). IVH is observed in 25% of infants with PVL and worsens WMI by increasing the amount of iron that combines with harmful free radicals and inflammatory cytokines to exacerbate injury (171, 172). The incidence of IVH has declined with current neonatal intensive care practices, but it remains an important problem, for which there is no targeted treatment (170). A recent paper demonstrated that intraventricular administration of hUCB–MSCs attenuates brain damage after severe IVH in newborn rats. The anti-inflammatory effects of MSCs (i.e., reducing the expression of inflammatory cytokines, such as IL-1α, IL-1β, IL-6, and TNF-α) were hypothesized to contribute to the prevention of ventricular dilation and neuroprotection (121). In adult rats, IV administration of hUCB–MNCs also showed amelioration of neurologic deficits associated with intra-cerebral hemorrhage (173).

## Oligodendrocytes, Myelin Deficits, and Umbilical Cord Blood

Loss of pre-oligodendrocytes and hypomyelination are the principal characteristics of preterm WMI and therefore protection of oligodendrocyte lineage cells must be central to the development of any neuroprotective strategies for the preterm brain. Recently, Zhu and colleagues have shown that hUC–MSCs increase mature oligodendrocyte number and improve long-term functional outcomes following hypoxia–ischemia in postnatal day 3 rats, with engraftment of cells at lesion sites (174). It will, however be important to further elucidate whether UCB stem cells can reduce pre-oligodendrocyte injury and thereby restore myelination in fetal or neonatal animal models of WMI. There is some indirect evidence that UCB cell populations may infer benefit to oligodendrocytes and myelination. The rare but serious genetic disorders termed leukodystrophies cause degeneration of myelin and progressive neurological deterioration and, to date, the only known treatment option for leukodystrophies is early transplantation of HSCs (175). Experimentally, spontaneous myelin mutants have been used to study potential therapies. The most commonly used myelin mutant in transplant experiments is the shiverer mouse, which has a mutation in the myelin basic protein gene, and has been extensively used to study myelination by exogenous cell transplantation, including HSCs, MSCs, and oligodendrocytes progenitor cells (OPCs). OPCs can be isolated, differentiated, and expanded from both fresh and cryopreserved UCB (176, 177) offering a potential treatment option, and lay the foundations for future studies in this research field.

## Clinical Trials for Cerebral Palsy

There are currently a number of clinical trials listed, or recently completed, for treatment of children with established CP. A pilot study from Hanyang University Medical Center, Republic of Korea examined 20 children aged 2–10 years with clinical CP, who were born either preterm or term, and administered peripheral autologous UCB–MNCs. Neurodevelopmental outcomes and neuroimaging studies were conducted up to 24 weeks after UCB administration, and compared with a pre-infusion baseline. Functional improvements were demonstrated in 25% of patients, and improvements in brain imaging outcomes were also noted in children with neurodevelopmental recovery. Side effects were identified in 25% of participants during infusion, treated successfully with antihistamines and hydration. Although not powered to demonstrate statistical benefit, the study showed the potential and safety of autologous UCB–MNC treatment in children (178). Between 2009 and 2012, Duke University in the USA treated 23 term newborns identified with HIE soon after birth with autologous UCB administration. The study was able to demonstrate feasibility and safety of autologous UCB re-administration in combination with hypothermia, targeting UCB administration at 6 h. Neurodevelopmental outcomes were recorded at 1 year of age, however, a greater number of babies will be required to appropriately assess functional outcomes (179). Duke University is continuing to recruit for a larger study of autologous UCB administration for children with established spastic CP.

## Use and Efficacy of Combination Therapies

A recent clinical study has assessed allogeneic UCB administered in combination with erythropoietin (EPO – itself the subject of neuroprotective trials), cyclosporine (an immunosuppressant), and rehabilitation therapy at Bundang CHA Hospital, Republic of Korea. Ninety-six children with CP aged 10 months to 10 years were treated with UCB cells, and improved cognitive and motor function were observed in all groups, including placebo, but with greater improvements in UCB + EPO children (180). Due to its design, this study does not separate the neuroprotective effects of EPO from UCB. However, combination therapy, which targets different mechanisms and therapeutic windows, may be a useful approach to treat preterm infants because of their multifactorial causes of injury and the inherent difficulties with identifying a therapeutic time frame in these infants. Indeed, ganglioside and mannitol have been shown to enhance the neuroprotective benefits of hUCB–MNCs and hUC–MSCs treatments following neonatal asphyxia in pre-clinical studies (130, 138). In contrast, despite recent promising neuroprotective outcomes of EPO in preterm cohort (181), routine clinical use of EPO, especially by high-dose, has always been hampered by its risk for retinopathy of prematurity (182). Melatonin, a powerful antioxidant shown to protect the developing brain by reducing oxidative stress following hypoxia–ischemia, with an absence of side effects, may be a candidate for co-administration with UCB (183). In term infants with HIE, hypothermia has been standard neuroprotective therapy for a number of years (184), and combination treatment of moderate hypothermia with MSCs significantly improves neuronal survival and mitochondrial activity after OGD exposure *in vitro* (185). Clinically, Cotten and colleagues recently showed that hypothermia and autologous UCB combination treatment is feasible and safe in term infants with HIE (179), and reflects that any treatment for term HIE must be considered in the context of therapeutic hypothermia. However, in very preterm and extremely preterm infants hypothermia is not currently recommended and may increase the risk of complications or death (186). A phase 1 clinical study of selected head cooling for preterm infants, born 32–35 weeks gestation, with neonatal HIE has recently been completed [NCT00620711], and the results are awaited with interest.

Other types of stem and stem-like cells may also be used for the combination therapy with UCB. UC, in addition to UCB, provides an abundant and non-invasive source of MSCs. These cells are neuroprotective in hypoxic-ischemic brain injury, and share similar *in vitro* immunosuppressive properties with bone marrow- and UCB-derived MSCs as well as mediating monocyte function to suppress T cell proliferation (130, 187). Importantly, hUC–MSCs also protect oligodendrocytes, reduce astrogliosis, and improve long-term functional outcomes in a model of preterm postnatal day 3 rats hypoxia–ischemia (174). Moreover, hUC–MSCs undergo successful cell expansion using animal serum-free culture medium, thereby removing safety concerns of animal-to-human viral transmission, further encouraging their potential for clinical application (188, 189). hAECs may present a useful therapy in combination with UCB, hypothermia, or alternate therapies. The proven anti-inflammatory properties of hAECs appear a principal mechanism to reduce preterm brain injury (88). They display both embryonic and pluripotent stem cells with abundant quantity, do not express MHC class molecules so have low immunogenicity, and do not form teratomas (190). Furthermore, the ready availability of hAECs without the need of expansion may enable them to be used for early autologous transplantation for preterm brain injury (88, 188, 189), with or without combination therapies.

## Optimal Timing of Transplantation of Umbilical Cord Blood

Recent experimental studies have been aimed at identifying the therapeutic window for UCB therapy. In adult rats who underwent middle cerebral artery occlusion-induced stroke, intravenous administration of hUCB–MNCs within 72 h resulted in an early functional recovery with lesion improvement, however cell administration at 120 h provided only minor functional recovery, and treatment at 14 days did not show any benefit (132). Whether a similar result can be obtained in an autologous or allogeneic setting is unknown. However, given that one of the primary benefits of UCB cells is their anti-inflammatory actions, it is likely that early intervention may be of greater benefit. Indeed, current ongoing clinical trials for neonatal HIE by National University Hospital, Singapore, and Duke University, USA are giving autologous UCB within the first 3 and 14 days, respectively after term birth asphyxia (NCT01649648 and NCT00593242). However, in preterm infants, it is difficult to know the timing of WMI that results in cystic PVL or diffuse WMI (9, 184, 191) and therefore either combination therapies, or cell preparations with multiple benefits would be most appropriate.

## Clinical Trials for Preterm Brain Injury

No trials of neuroprotective UCB for use in treating WMI in preterm infants are currently registered in humans. A significant challenge in the design of a clinical trial for preterm infants is the question of which UCB cells to administer? As described above, it is becoming apparent that the type and quantity of specific cell types differs in preterm UCB from that in term UCB. A dose–response effect of UCB therapy for neonatal hypoxia–ischemia has been demonstrated (133, 192), but it is unclear whether a therapeutic quantity of cells can be derived from preterm UCB as the volumes obtained are low (see Umbilical Cord Blood above). Further to this, the ability to expand preterm UCB cells is not yet well described.

In preterm infants, as also discussed above, the timing of the onset, and chronic progression of WMI is usually not known. It is also not known whether to administer UCB cells before or after brain injury is identified. Administration following the identification of brain injury may provide better outcomes than administration in later childhood, due to the plasticity of the developing brain; although the evidence for this both in pre-clinical studies and clinical trials is sparse. In contrast, as cystic PVL or diffuse WMI tend to develop over days to weeks after birth (72, 169), early postnatal UCB administration “before defining brain damage” in preterm and extremely low birth weight infants may be more efficacious. Indeed, two clinical trials administering autologous UCB to preterm infants in the first 5 or 14 days post-delivery, aiming to examine feasibility and efficacy for a variety of preterm complications, are currently underway (NCT02050971 and NCT01121328). However, in preterm infants susceptible to WMI at least until 32 weeks or more gestational age, a single administration of cells might not span an adequate period of brain protection. Thus, the need for repeated dose administration and expansion of UCB samples are further exemplified in a preterm cohort. Recently described *in vivo* cell tracking methodology using MRI, which enables the tracking of migration and distribution of magnetically labeled cells in tissues, may be useful for optimizing the time course for UCB treatment (193).

## Conclusion

Taking into account the similarities and differences in preterm versus term brain injury, and limitations to date in stem cell studies for preterm WMI, it is apparent that a number of considerations apply before UCB treatment could be extended to infants born preterm. The clear advantage of undertaking UCB administration in a preterm cohort is the relative plasticity of the developing brain in immature infants, and potential for regeneration. However, there are current disadvantages that must be overcome. Studies to date suggest that early cell administration post-injury achieves favorable therapeutic outcomes, but a current lack of sensitive diagnostic tools and inability to accurately determine the onset of preterm brain injury remains problematic. Work to define the most appropriate time for therapeutic intervention is needed. Additionally, the specific cells present in UCB responsible for brain protection are not yet characterized and the complications of pregnancy that are often co-morbidities with preterm birth, such as uteroplacental inflammation or IUGR, may alter the cellular composition of UCB. A handful of published work suggests that preterm UCB cell number, cell total population and cell maturity is different to that in term UCB, which may not provide the expected benefit that has been observed using term hUCB in experimental animal and clinical studies. It is therefore currently not known whether autologous or allogeneic UCB cell administration would confer optimal benefit in a preterm cohort, or whether expansion of specific cell types should be considered and pursued. Thus it remains that UCB holds strong promise for the treatment of preterm brain injury in the neonate, but fundamental questions must be answered with appropriately designed experimental animal and clinical studies prior to large-scale randomized clinical trials for preterm brain injury.

## Conflict of Interest Statement

The authors declare that the research was conducted in the absence of any commercial or financial relationships that could be construed as a potential conflict of interest.
